# Identification and Integrative Discovery of Anti-Inflammatory Compounds Isolated from *Eclipta prostrata* (L.) L. by Network Pharmacology, Molecular Docking, and In Vitro Evaluation

**DOI:** 10.3390/ph18111653

**Published:** 2025-11-01

**Authors:** Cao Van Anh, Nguyen Ngoc Linh, Phuochien Phan

**Affiliations:** 1 Institute of Pharmaceutical Education, Vietnam Military Medical University, Hanoi 100000, Vietnam; caovananh12a1@gmail.com; 2 Institute of Medicine and Pharmacy, Thanh Do University, Hoai Duc, Hanoi, Vietnam; nnlinh@thanhdouni.edu.vn; 3 Institute of Applied Science and Technology, Van Lang School of Technology, Van Lang University, Ho Chi Minh City, Vietnam; 4 Faculty of Applied Technology, Van Lang School of Technology, Van Lang University, Ho Chi Minh City, Vietnam

**Keywords:** *Eclipta prostrata*, isolated compounds, network pharmacology, molecular docking, inflammation

## Abstract

**Background/Objective**: *Eclipta prostrata* (L.) L. is a traditional medicinal herb utilized throughout Asia that is widely used for hepatoprotective activity, wound healing, and blood cooling/bleeding disorders. This work aimed to identify bioactive constituents from *E*. *prostrata* collected in Vietnam, and clarify their anti-inflammatory capacity of the extract and active fraction. **Method**: Extraction and isolation of compounds from the extract of *E*. *prostrata* were performed. The extract, fractions, and isolated compounds were evaluated for inflammatory cytokines in LPS-stimulated RAW264.7 cells. Isolates showed inflammatory potential by in silico approaches. **Results**: Thirteen compounds, comprising a first isolated compound (diosmin), flavonoids, and phenolic derivatives, were separated and identified. The protein–protein interaction (PPI) network demonstrated TNF, IL6, AKT1, NFKB1, EGFR, and PTGS2 as central targets, highlighting their significance in inflammatory signaling. Gene Ontology and KEGG pathway enrichment underscored substantial participation in TNF and IL-17 cytokine signaling pathways. Molecular docking demonstrated robust interactions between several flavonoids and core targets, indicating their function as essential regulators. Experimental validation in LPS-stimulated RAW264.7 macrophages revealed that wedelolactone, luteolin, apigenin, and quercetin significantly inhibited TNF-α and IL-6 production. **Conclusions**: The results proposed that *E*. *prostrata* demonstrates its anti-inflammatory efficacy via a multi-target, poly-pharmacological strategy that encompasses central cytokine pathways and upstream receptor-mediated signaling. Our findings offer new mechanistic evidence that supports the ethnomedicinal application of *E*. *prostrata* and indicates its potential as a valuable natural resource for treating anti-inflammatory diseases.

## 1. Introduction

*Eclipta prostrata* (L.) L., belongs to the family Asteraceae distributed across tropical and subtropical regions worldwide [1]. This plant has a long history of use in traditional healing systems across Asia, Africa, and South America. In Ayurveda, it is classified as a “rasayana” drug and used to rejuvenate the body, promote hair growth, and treat hepatic and skin disorders. In Traditional Chinese Medicine, it is prescribed for cooling, detoxification, and the management of bleeding disorders, hepatitis, and infectious diseases. Folk remedies also employ this plant for wounds, snakebites, respiratory ailments, and gastrointestinal disturbances. Previous studies revealed chemical constituents including coumestans (wedelolactone, demethylwedelolactone), flavonoids (luteolin, apigenin, quercetin), triterpenoids, and sterols as its major constituents [2,3]. Moreover, pharmacological studies displayed a broad spectrum of activities, including hepatoprotective, antioxidant [4], antimicrobial, antiviral [5], anticancer [3], and antidiabetic [2] effects, which scientifically validate many of its traditional uses. This dual ethnomedicinal and pharmacological evidence underscores the therapeutic relevance of *E*. *prostrata* and supports its continued exploration as a source of novel bioactive compounds.

Inflammation is a highly conserved biological responses of the immune system to harmful stimuli such as pathogens, tissue injury, or metabolic stress [6]. Under normal conditions, acute inflammation is essential for eliminating infectious agents, repairing damaged tissues, and restoring homeostasis [7]. However, when inflammation becomes chronic, excessive, or unresolved, it contributes to the pathophysiology of a diverse range of human diseases, including autoimmune disorders, metabolic syndromes, neurodegenerative diseases, cardiovascular diseases, and cancer [8]. The dual nature of inflammation as protective in the short term but pathogenic when dysregulated has made it a central focus in biomedical research. This process is mediated by a complex interplay of signaling pathways, notably nuclear factor kappa B (NF-κB), mitogen-activated protein kinase (MAPK), Janus kinase (JAK)/signal transducer and activator of transcription (STAT), phosphoinositide 3-kinase (PI3K)/protein kinase B (AKT), and inflammasome activation, which regulate the expression of cytokines, chemokines, and inflammatory mediators such as tumor necrosis factor-alpha (TNF-α), interleukin-6 (IL-6), and cyclooxygenase-2 (COX-2). Among these, IL-6 and TNF stand out as key pro-inflammatory cytokines, with IL-6 driving the transition from acute to chronic inflammation, and influencing leukocyte infiltration [9], while TNF induces inflammation and promotes cell death [10]. Prostaglandin–endoperoxide synthase 2 (PTGS2)/COX-2, upregulated in response to tissue damage, further amplifies the inflammatory cascade through prostaglandin production, impacting cell survival pathways and immune cell recruitment [11]. Epidermal growth factor receptor (EGFR) signaling emerges as a regulator of macrophage responses to infection, particularly in the gut, suggesting its potential as a therapeutic target for chronic gut inflammation. Intracellularly, AKT1 plays a multifaceted role in cell metabolism, survival, and migration during inflammation, modulating vascular permeability, and leukocyte emigration into inflamed tissues [12]. Finally, nuclear factor of kappa light polypeptide gene enhancer in B-cells 1 (NFKB1), a crucial transcription factor, regulates the expression of numerous genes involved in inflammation and immune responses, acting as both an activator and, under certain conditions, a suppressor of inflammation [13]. Understanding the intricate interplay of these molecules is crucial for developing targeted therapies to modulate inflammatory responses in various disease states. The dysregulation of these factors contributes significantly to the pathogenesis of autoimmune disorders, chronic inflammatory conditions, and even cancer, highlighting their potential as therapeutic targets. Consequently, targeting inflammatory signaling cascades has emerged as a critical therapeutic strategy, with both synthetic drugs and natural products being investigated for their potential to modulate key regulators of these pathways.

This study discovers the anti-inflammatory capacities of extract and fractions, and the separation of 13 compounds from ethanolic extract of *E*. *prostrata* collected in Vietnam through applying multiple chromatographic methods, including silica gel, octadecylsilane (ODS), and Sephadex column chromatography. The structures of these isolated compounds were identified by using nuclear magnetic resonance spectroscopy (NMR) and mass spectroscopic methods. The isolated compounds were investigated for their anti-inflammatory potential by utilizing network pharmacology and molecular docking in silico approaches to identify the hub genes of compounds in inflammation, and determine the potential mechanisms in inflammation. Isolated compounds were further evaluated by their anti-inflammatory properties by examining the inhibitory effect on TNF-α and IL-6 production induced by lipopolysaccharides (LPS)-stimulated RAW264.7 cells.

## 2. Results

### 2.1. Anti-Inflammatory Effect of Extract and Fractions

*E. prostrata* extract (Ext) and fractions [n-hexane(H), EtOAc (E), water (W)] were examined for their inhibition against IL-6 and TNF-α production secreted by LPS-activated RAW264.7 cells. The results indicated that the E fraction significantly inhibited the production of inflammatory cytokines, with half-maximal inhibitory concentrations (IC_50_) of 43.24 ± 0.83 μg/mL and 45.97 ± 2.01 μg/mL against IL-6 and TNF-α production, respectively, without exerting cytotoxic effects on cell viability (Table S1, Supplementary Materials). Thus, this fraction was selected for the separation of active constituents (Table 1).

### 2.2. Separation and Identification of Compounds from the Most Active Fraction

**Figure 1:**
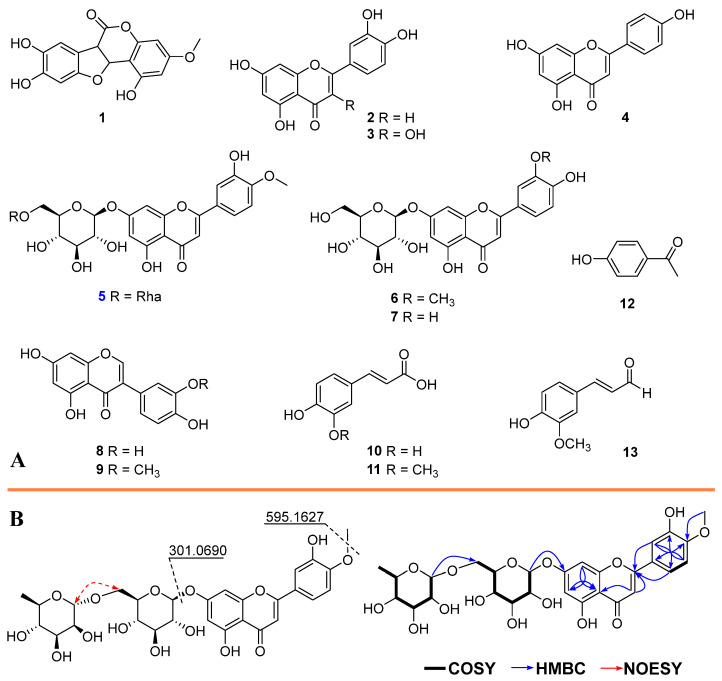
Chemical structures of isolated compounds (**1**–**13**, (**A**)) and key COSY, HMBC, and NOESY correlations of compound **5** (**B**).

To characterize the chemical constituents responsible for the bioactivity, the E fraction was subjected to successive chromatographic separations using a series of mobile phase systems composed of mixed organic solvents, in conjunction with diverse stationary phases, including normal-phase and reversed-phase silica gel, as well as Sephadex LH-20. These purification procedures ultimately yielded thirteen compounds (Figure 1A). Among isolated compounds, compound **5** was isolated as a minor constituent with a light-yellow powder. Its high-resolution electrospray ionization mass (HR-ESI-MS) spectrometry showed a protonated ion peak at *m*/*z* 609.1782 (calculated for C_28_H_33_O_15_, 607.609.1819). At the same time, a high mass spectrum of **5** also displayed fragments at *m*/*z* 595.1627 and 301.0690, characterized for [M+H-CH_3_]^+^ and for [M+H-Glc-Rha]^+^, indicated for a reduction of a methyl group and reduction of glycosides of **5**, respectively. The proton nuclear magnetic resonance (^1^H NMR) spectrum of **5** showed the characteristics of flavonoid backbone including an ABX spin system at *δ*_H_ 7.57 (1H, dd, *J* = 8.8, 2.3 Hz, H-6′), 7.45 (1H, d, *J* = 2.3 Hz, H-2′), and 7.13 (1H, d, *J* = 8.8 Hz, H-5′), typical meta-coupling signals at *δ*_H_ 6.76 (1H, J = 8.8, 2.3 Hz, H-8) and 6.46 (1H, J = 8.8, 2.3 Hz, H-6), and an olefinic singlet at *δ*_H_ 6.82 (1H, H-3) (Table S1, Supplementary Materials). The carbon-13 nuclear magnetic resonance (^13^C NMR) spectrum of **5** showed the deshelled signals of conjugated-oxygen carbons at *δ*_C_ 164.2 (C-2), 182.0 (C-4), 161.2 (C-5), 162.9 (C-7), and 156.9 (C-9), together with quaternary carbon at *δ*_C_ 105.5, revealed a typical of a fused ring system. An anomeric proton resonated at *δ*_H_ 5.08 (1H, *J* = 7.4 Hz, H-1′), consisted with *β*-D-glucose.

Additionally, another anomeric resonated at *δ*_H_ 4.54 (1H, *J* = 1.7 Hz, H-1″), revealed for α-L-rhamnose. The heteronuclear multiple-bond correlation (HMBC) showed a long-range correlation between a methyl group at *δ*_H_ 3.87 and C-4′ (*δ*_C_ 151.3), indicated for 4′-OCH_3_ attachment. HMBC spectrum also displayed a long-range correlation from H-1′ (*δ*_H_ 5.08) to C-7 (*δ*_C_ 162.9), and those of H-8 (*δ*_H_ 6.76) to C-7 (*δ*_C_ 162.9)/C-9 (*δ*_C_ 156.9), confirmed 7-*O*-Glc combination (Figure 1B). Moreover, a HMBC long-range correlation between H-1″ (*δ*_H_ 4.54) and C-6′ (*δ*_C_ 66.1), suggested the second rhamnose attached to C-6 of the first sugar unit. This observation explained the upfield shift at C-6″ (*δ*_C_ 66.1). Finally, **5** was elucidated as diosmin. This is the first report on the identification of this compound from *E*. *prostrata*.

Moreover, an additional 12 compounds (**1**–**4** and **6**–**13**) were also identified. Their structures were identified by interpreting their spectroscopic data comparing to those reported in the literature, including wedelolactone (**1**) [14], luteolin (**2**) [2], quercetin (**3**) [15], apigenin (**4**) [2], chrysoeriol 7-*O*-*β*-D-glucoside (**6**) [16], luteolin 7-*O*-glucopyranoside (**7**) [17], orobol (**8**), pratensein (**9**) [2], caffeic acid (**10**) [18], ferulic acid (**11**) [19], 4′-hydroxyacetophenone (**12**) [3], and coniferylaldehyde (**13**) [20].

### 2.3. Anti-Inflammatory Potential of Isolated Compounds of E. prostrata

#### 2.3.1. Construction of the PPI Network and Core Targets

**Figure 2:**
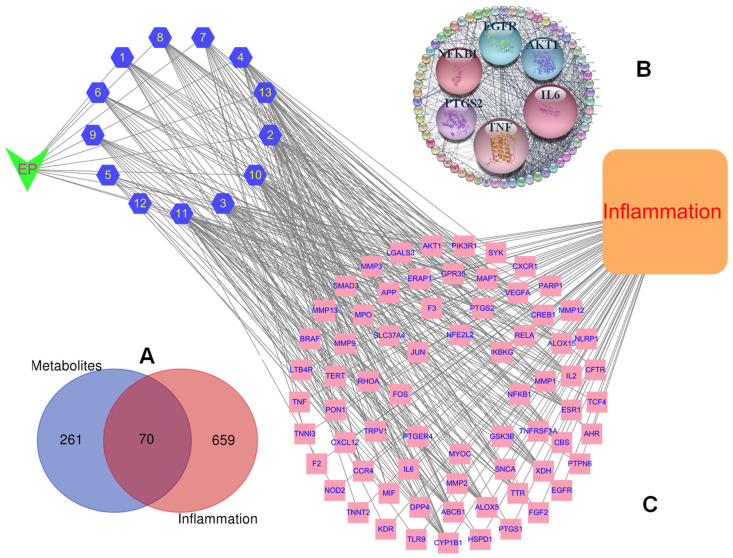
(**A**) Venn diagram showing the overlapping targets between compound-related genes and inflammation-associated genes, with the intersecting region representing potential core targets. (**B**) PPI network of the targets, where hub nodes indicate key regulators in inflammatory signaling. (**C**) Compound–target–inflammation network illustrating multitarget interactions between bioactive compounds and inflammation-related proteins, highlighting the poly-pharmacological nature of the compounds: *E. prostrata*, compounds, and targets representing as V angle, hexagon, rectangle, respectively.

To evaluate the pharmacological actions of isolated compounds from *E*. *prostrata* against inflammatory diseases, a PPI network was constructed by mapping target interactions to identify key hub proteins that play central roles in biological processes and signaling pathways [21]. The SMILES formats of isolated compounds were imported to the SEA database, identifying 331 protein targets associated with 13 constituents by using screening criterion (Possibility > 0), and followed by consolidating and removing duplicates. To refine disease relevance, 729 inflammation-related targets were also obtained from the GeneCards database [22] after screening thresholds (Score > 5) and removing duplicates. As a result, 70 intersecting targets represented as purple circles were identified by using the Venn diagram [23] (Figure 2A) for subsequent analysis. This observation suggested that compounds isolated from *E*. *prostrata* may exhibit anti-inflammatory effects as their predicted targets overlap with known inflammation-related proteins. Subsequently, these intersecting targets were loaded into the STRING database for building the protein–protein interaction (PPI) network (Figure 2B) using the STRING plugin Cytoscape (Ver. 3.10) software, composing of 68 nodes (after removing two unconnected nodes) related to 700 action targets as edges. The network exhibited a high average number of neighbors (20.588), a short characteristic path length (1.747), and a high clustering coefficient (0.689), indicating dense connectivity and modular organization of intersecting targets. The PPI network revealed key inflammation regulators (IL6, TNF, AKT1, NFKB1, EGFR, and PTGS2) as central nodes. These findings suggested that the compounds are likely modulated multiple inflammatory pathways rather than targeting a single protein.

High degrees of the hub genes, [IL6 (60), TNF (59), AKT1 (52, NFKB1 (47), EGFR (46), PTGS2 (44)], indicated a high level of connectivity, suggesting the influential role in the biological network. A full herbal–compound–target–inflammation network revealed that all compounds connect through multiple targets that ultimately converge on the disease node “Inflammation,” showing a multi-compound, multi-target, single-disease framework (Figure 2C). This network illustrated the interactions of compounds derived from *E*. *prostrata* that target inflammation, highlighting the principal targets and essential metabolites for inflammation treatment and potential of anti-inflammatory effects through a poly-pharmacological mechanism, where isolated compounds simultaneously modulate multiple targets involved in the disease process.

#### 2.3.2. Enriched Gene Ontology (GO) Clusters

To investigate the biological mechanisms of action, top 10 GO enrichment results showed that the most enriched processes are cellular response to chemical stimulus, responses to chemical and organic substances [24], and response to stress, which are important for inflammatory responses triggered by cytokines, LPS, or reactive oxygen species (ROS) [25].

**Figure 3:**
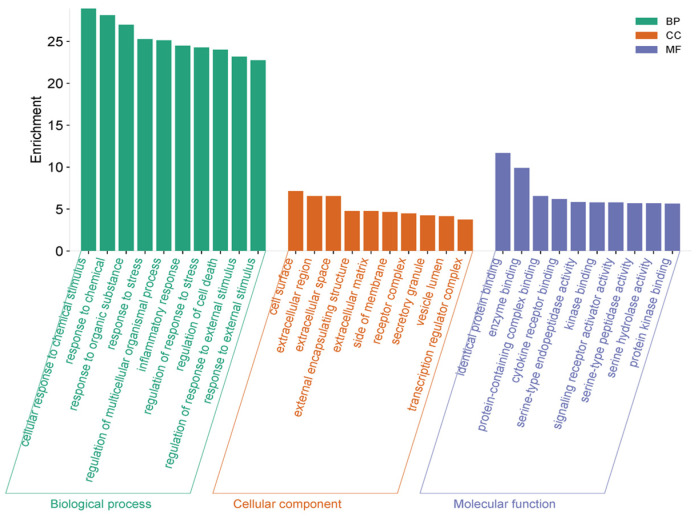
GO enrichment analysis of potential targets for anti-inflammatory improvement. Biological processes (BP), cellular components (CC), and molecular functions (MF) are represented as green, orange, and blue color, respectively.

In addition to the above findings, the bioactive compounds isolated from the active fraction affect pathways that control cytokine secretion, immune cell recruitment, and oxidative stress defense regulation of inflammatory response and regulation of response to stress–biological processes (Figure 3). Targets are enriched in cell surface, extracellular region, and extracellular matrix, which are the primary sites for cytokine–receptor interaction and immune cell communication. Enrichment inside of a membrane and receptor complex indicates the regulation of receptor-mediated signaling, crucial in initiating inflammatory cascades. Thus, this suggests that *E*. *prostrata* compounds act by modulating extracellular signaling and receptor-ligand interactions rather than only intracellular metabolism. Additionally, top enriched functions include identical protein binding, protein-containing complex binding, and enzyme binding, which reflect protein–protein interactions involved in signal transduction. The GO enrichment results highlight that the anti-inflammatory effects of *E*. *prostrata* are mediated by regulating stress and inflammatory responses (BP), mainly through extracellular and membrane-associated targets (CC), which are involved in cytokine–receptor interactions and receptor signaling activation (MF). This strongly suggests that the extract modulates early upstream events in inflammation, such as receptor-mediated cytokine signaling and oxidative stress response, while also influencing downstream processes like apoptosis and protease activity. Altogether, these enriched GO terms indicate that *E*. *prostrata* compounds exert broad anti-inflammatory potential by blocking extracellular inflammatory signaling, reducing cytokine activity, and regulating stress-induced cellular responses.

#### 2.3.3. Prediction of Potential Therapeutic Targets by the Kyoto Encyclopedia of Genes and Genomes (KEGG) Enrichment

**Figure 4:**
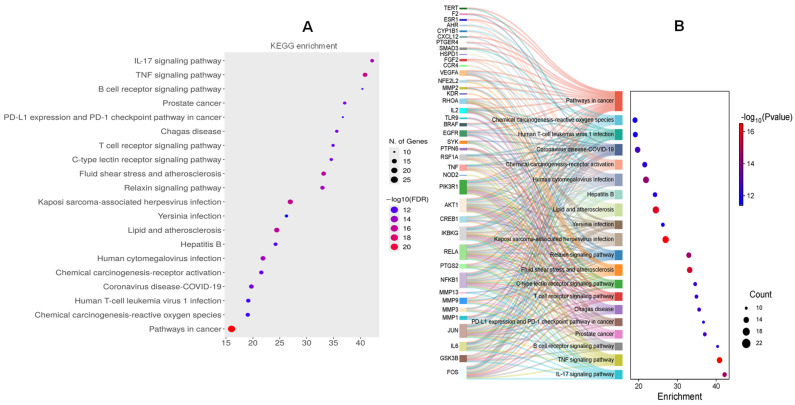
(**A**) Top 20 KEGG pathway enrichment of target genes. (**B**) Sankey plot on the left represents the core targets included in each pathway, and the conventional bubble plot is on the right. Bubble size indicates the number of potential targets to which the pathway belongs, and the bubble color indicates the *p*-value.

KEGG enrichment analysis revealed 191 related pathways. According to the enrichment score, the top 20 pathway was sorted (Figure 4A), *E*. *prostrata* compounds mainly target pro-inflammatory cytokine pathways (IL-17, TNF), immune receptor-mediated signaling (B/T cell, lectin receptors), and infection-related inflammation pathways, while also intersecting with chronic inflammatory diseases (atherosclerosis, ROS signaling) and cancer-related inflammation. This strongly indicates that these compounds exert multi-level anti-inflammatory effects by suppressing cytokine signaling, regulating immune cell activity, reducing oxidative stress, and potentially restoring immune regulatory balance.

Among inflammatory pathways, IL-17 and TNF signaling pathways are the most significantly enriched, both of which are central regulators of chronic and acute inflammation (Figures S2 and S3, Supplementary Materials). Here, IL-17 drives neutrophil recruitment, cytokine secretion (IL-6, GM-CSF), and tissue inflammation, and TNF signaling mediates NF-κB activation, apoptosis, and pro-inflammatory cytokine release. Furthermore, the Sankey map (Figure 4B) showed that immune inflammatory signaling pathways (TNF, IL-17, B/T cell receptor signaling) are highly enriched, with core targets (IL6, TNF, PTGS2, NFKB1) acting as hubs. Among them, the TNF signaling pathway interacted with all core targets. The present network analysis emphasizes the central involvement of TNF-α and IL-6 in mediating the inflammatory responses targeted by the compounds identified from the extract. Both cytokines exhibited high degree centrality in the PPI network, suggesting that they act as major hubs connecting multiple signaling pathways relevant to inflammation. KEGG enrichment analysis identified specific signaling and disease pathways, direct manifestations, and mechanistic details of the identified GO terms, with a strong focus on immune and inflammatory cascades.

### 2.4. Molecular Docking Analysis

**Figure 5:**
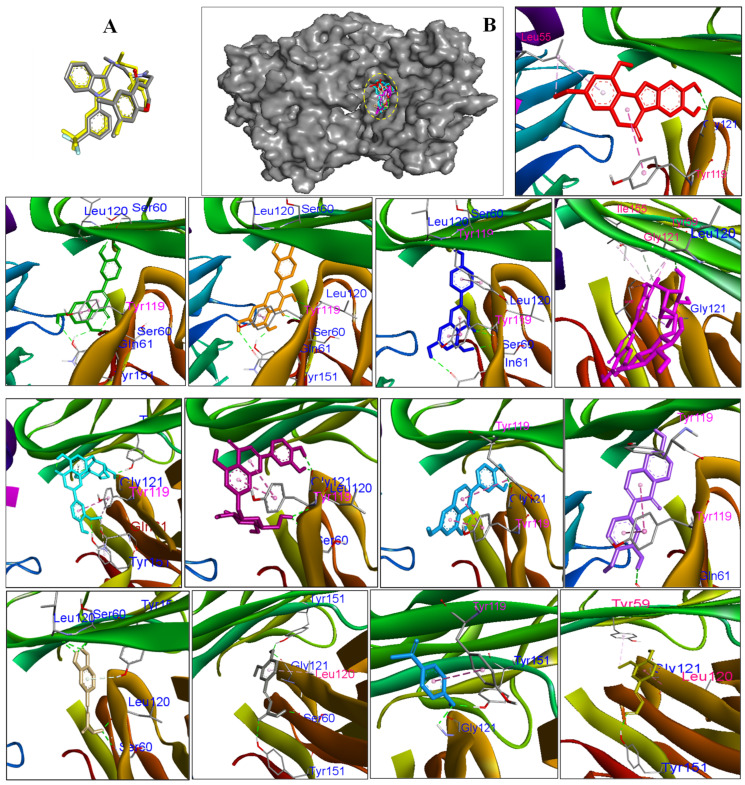
(**A**) The original and redocked native ligand were superimposed. (**B**) Compounds **1**–**13** were docked into the protein binding site. The TNF protein residues interact with compounds **1** (red), **2** (green), **3** (orange), **4** (blue), **5** (magenta), **6** (cyan), **7** (wheat), **8** (light blue), **9** (purple), **10** (sand), **11** (grey), **12** (marine), and **13** (limon). Hydrogen bonds between the ligand and residues are represented by blue characteristics.

To validate the interactions between isolated compounds and inflammation-related targets predicted by network pharmacology analysis, we employed a structure-based approach involving molecular docking. This facilitated the quantitative assessment of binding affinities and investigation of molecular interaction patterns. The top six core target proteins were then subjected to molecular docking with the isolated compounds to evaluate their binding affinities. These compounds showed scoring function values ranging from −8.01 to −3.45 kcal/mol toward core targets such as IL6, TNF, AKT1, NFKB1, EGFR, and PTGS2 (Table 2), suggesting potential molecular interactions relevant to inflammation regulation. Among them, compounds (**1**–**4**) demonstrated the low docked scores to the core targets compared to the other compounds. Particularly, compounds (**1**–**4**) showed stronger binding affinity to both top 2 targets, IL6 and TNF proteins (Table 2). This observation suggests that the isolated compounds may potentially contribute to the observed anti-inflammatory activity by modulating key targets involved in cytokine regulation and inflammatory pathways. Notably, compound **3** exhibited the strongest binding affinity with a score of –6.20 kcal/mol to TNF protein (Figure 5), suggesting a highly favorable interaction with the target site.

This was followed closely by compounds **1**, **2**, and **4** with docked scores of −5.01, −5.65 and −5.63 kcal/mol, respectively. Hydrogen bonding was observed as a critical stabilizing force across the ligand–receptor complexes [26]. These compounds demonstrated extensive hydrogen bonding networks involving residues such as Ser60, Gln61, Tyr119, LeuA120, Gly121, and Tyr151, which are key polar residues linking the binding pocket [27]. Additionally, they also displayed the hydrophobic interaction with Tyr 119 by forming π–π stacking and π–alkyl interactions. Additionally, compound **5** also displayed a low dock score of −5.47 kcal/mol through forming hydrogen bonds with LeuA120 and Gly121 residues at the binding sites of protein. Similarly, compounds (**1**–**4**) also display interactions with the key amino acids of IL6 proteins by forming hydrogen bonds with Leu33, Glu51, His 164, Arg168, and Lys171 of the protein (Figure S4, Supplementary Materials). Molecular docking analysis revealed key interactions between the isolated compounds and target proteins AKT1, NFKB1, EGFR, and PTGS2. For AKT1, compounds **1**, **2**, **4**, and **6** docked into the PH domain [28], and formed hydrogen bonds with Arg48 and Glu49. Compounds **4**, **6–8** interacted with Lys30 and Arg48 via hydrogen bonds (Figure S5, Supplementary Materials). Docking with NFKB1 showed that compounds **2**–**4**, **6**–**10**, **12**, and **13** formed hydrogen bonds with Arg35, while compounds **6**–**8**, **10**, **11**, and **13** interacted with Arg33 [29] via hydrogen bonds (Figure S6, Supplementary Materials). Similarly, for EGFR, compounds **2**–**4**, **6**, **7**, **9**, and **13** interacted with Gln791 through hydrogen bonds, and compounds **1**–**4**, **7**–**11** interacted with Arg776 [30] (Figure S7, Supplementary Materials). Finally, docking with PTGS2 showed that compounds **2**, **3**, **4** interacted with Tyr355, Met522, and Ser530 via hydrogen bonds [31], while compounds **10**–**12** formed hydrogen bonds with Asn375 (Figure S8, Supplementary Materials).

### 2.5. Anti-Inflammatory Effects of Isolated Compounds (**1**–**13**) In Vitro

To verify the anti-inflammatory potential of isolated compounds (**1**–**13**), we were evaluated for their inhibition to TNF-α production induced by LPS-activated RAW264.7 cells. Among them, compounds **2**–**4** strongly suppressed TNF-α production with IC_50_ values of 2.56 ± 0.08, 6.18 ± 0.03, and 8.01 ± 0.11 μM, respectively (Table 3).

Compound **9** moderately reduced TNF-α production (IC_50_ value: 12.30 ± 1.06 μM, respectively). Other compounds showed the weak or inactive effect under the experimental conditions. Furthermore, these isolated compounds also demonstrated inhibitory effects on IL-6 production secreted by LPS-activated RAW264.7 cells. Compounds (**1**–**3**) suppressed IL-6 production with IC_50_ values of 5.66 ± 0.54, 13.24 ± 0.73, and 9.89 ± 0.80 μM, respectively. Compounds **4**, **8**, and **13** also inhibited IL-6 production with IC_50_ values of 25.45 ± 1.26, 17.45 ± 1.30, and 23.81 ± 0.45 μM, respectively. Other compounds weakly suppressed IL-6 production induced by LPS-stimulated RAW264.7 cells. All tested compounds did not cause significant effect on cell viability under experimental conditions (Figure S9, Supplementary Materials). The results indicated that compounds **1**–**4** demonstrated the most pronounced anti-inflammatory effects compared to the LPS control (without compound addition).

## 3. Discussion

The extract and fractions of *E*. *prostrata* significantly affected the levels of cytokine production in LPS-activated RAW264.7 cells. The crude extract (Ext.) demonstrated intermediate inhibition. The E fraction markedly reduced both IL-6 and TNF-α production levels with IC_50_ values of 43.24 ± 0.83 µg/mL and 45.97 ± 2.01 µg/mL, respectively. The H and W fractions showed moderate and weak anti-inflammatory activity toward cytokine production. Therefore, the E fraction may serve as a promising candidate for further isolation and characterization of active anti-inflammatory compounds. The anti-inflammatory activity of the extract and fractions of *E*. *prostrata* guided the separation of a previously unisolated compound, diosmin, along with two flavonoid glycosides, four flavonoids, two isoflavonoids, and three phenolics derived from the ethanolic extract of the whole *E*. *prostrata* plant by using multiple chromatographic techniques. The structures of isolated compounds were successfully elucidated using multiple spectroscopies, mass analysis and compared to those reported from the literature. *E*. *prostrata* is a medicinal herb recognized for its diverse array of flavonoid and phenolic derivatives, many of which have been isolated and characterized through phytochemical studies. Previous studies also revealed the contents of phenolic and flavonoid derivatives from this plant responsible for multiple pharmacological actions [32,33]. Especially, the EtOAc fraction demonstrated the enrichment of the phenolic and flavonoid compounds through its high contents of these compounds [4,34]. Flavonoids from *E*. *prostrata* exhibited broadened bioactivities, including antitumor [3], antioxidant [4], hypoglycemic [35], and antidiabetic [2] activities. On the other hand, phenolic derivatives found in *E*. *prostrata* displayed antioxidant [34], anti-inflammatory [2] activities. A pro-inflammatory cytokine, tumor necrosis factor (TNF) is pivotal in the development of inflammatory diseases and in controlling immunological responses [36]. Initially identified for its ability to induce tumor cell apoptosis, TNF is now recognized as a key mediator of inflammation, contributing to both innate and adaptive immunity. Through its interaction with TNF receptors (TNFR1 and TNFR2), TNF activates diverse signaling cascades, including NF-κB and MAPK pathways, leading to the expression of adhesion molecules, chemokines, and other cytokines that amplify immune responses [10]. Many chronic inflammatory and autoimmune disorders, including psoriasis, ankylosing spondylitis, inflammatory bowel disease, and rheumatoid arthritis, are characterized by abnormal or excessive TNF production. In these conditions, TNF drives pathological inflammation, tissue destruction, and systemic manifestations. The clinical relevance of TNF in disease has been underscored by the development of TNF inhibitors, which have significantly improved patient outcomes and transformed the therapeutic landscape for many inflammatory disorders [37]. Despite its therapeutic targeting, the complex and context-dependent functions of TNF remain areas of active research. Understanding the molecular mechanisms governing TNF signaling and its interplay with other inflammatory mediators is essential for refining current treatments and developing novel interventions for TNF-associated diseases. IL-6 also displayed substantial connectivity within the network, underscoring its pivotal contribution to both acute and chronic inflammation. Unlike TNF-α, which primarily amplifies innate responses, IL-6 bridges innate and adaptive immunity. GO enrichment linked IL-6 to processes such as the positive regulation of JAK-STAT signaling and acute-phase response, reflecting its role in promoting hepatic acute-phase protein synthesis and Th17 differentiation. KEGG mapping placed IL-6 within the IL-17 signaling pathway and cytokine–cytokine receptor interaction pathway, highlighting its role in sustaining chronic inflammatory states. Excessive IL-6 production is implicated in autoimmune diseases and metabolic inflammation, suggesting that its modulation could mitigate systemic inflammatory responses and prevent long-term tissue damage. Although TNF-α and IL-6 act through distinct mechanisms, their interplay is critical in sustaining chronic inflammation. TNF-α induces IL-6 production in macrophages and other immune cells, forming a positive feedback loop that perpetuates inflammatory signaling. The co-enrichment of both cytokines across multiple GO terms and KEGG pathways in this study underscores their synergistic roles as hubs of the inflammatory interactome. From a therapeutic perspective, targeting both TNF-α and IL-6 simultaneously through multi-component extracts may achieve more effective modulation of inflammation than inhibition of a single cytokine alone. The identification of compounds with predicted interactions against these two cytokines, therefore, provides a mechanistic basis for the observed anti-inflammatory potential of the extract.

The isolated compounds (**1**–**13**) significantly alternated the IL-6 and TNF-α production secreted in LPS-activated RAW264.7 cells. Based on the inhibitory effect of these compounds, a structural activity relationship was deduced. Compounds **4** and **7** are apigenin derivatives. However, compound **4** is more active than **7**, thereby glucoside attachment may be not favor for anti-inflammatory effect under tested conditions. Compounds **2** and **4** showed a stronger suppression to TNF-α production secreted in LPS stimulated RAW264.7 cells than those of compounds **8** and **9**. Thereby, the flavonoid is more flavored to anti-inflammatory effect than those of iso-flavonoid under the same tested conditions. Previous studies have revealed that several flavonoids have been reported to regulate inflammatory responses through multi-target mechanisms, with TNF-α and IL-6 emerging as central mediators. Wedelolactone markedly reduced the IL-1β-induced expression of COX-2, iNOS, TNF-α, and IL-6 in human chondrocytes by inhibiting NF-κB activation and further attenuated colonic inflammation in DSS-induced colitis via suppression of the IL-6/STAT3 axis [38]. Luteolin displayed potent anti-inflammatory activity in both in vitro and in vivo models of arthritis, where it inhibited NO, PGE_2_, and TNF-α production, downregulated COX-2 and iNOS expression, and reduced matrix metalloproteinases through blockade of NF-κB and MAPK signaling [39]. Quercetin similarly attenuated inflammatory responses in LPS-stimulated macrophages by lowering IL-1β, IL-6, and TNF-α transcription and inhibiting MAPK and NF-κB signaling cascades [40]. Apigenin also demonstrated strong suppression of TNF-α-induced NF-κB activation and chemokine expression, while alleviating acute inflammatory reactions in vivo [41]. Collectively, these findings highlight that wedelolactone, luteolin, quercetin, and apigenin share overlapping anti-inflammatory mechanisms centered on the inhibition of NF-κB and MAPK pathways, ultimately leading to the downregulation of TNF-α, IL-6, and related cytokines, thereby reinforcing their therapeutic relevance in inflammation-associated diseases. Wedelolactone has been widely reported to exert potent anti-inflammatory effects through multi-target regulatory mechanisms. In vitro studies demonstrated that wedelolactone decreased the expression levels of TNF-α, iNOS, IL-6, and COX-2 in LPS-stimulated macrophages and IL-1β-treated chondrocytes, largely via inhibition of NF-κB activation and stabilization of IκB-α [42,43]. In animal models, wedelolactone alleviated DSS-induced colitis by blocking NF-κB and MAPK pathways, suppressing NLRP3 inflammasome activation, and promoting AMPK signaling, thereby reducing IL-1β release and tissue injury. Similarly, in models of doxorubicin-induced podocyte injury and collagen-induced arthritis, wedelolactone downregulated IL-6, MCP-1, TNF-α, TGF-β1, and IL-18, while attenuating oxidative stress and synovial inflammation [44]. This study collectively illustrates that the isolated compounds contribute to the anti-inflammatory efficacy of *E*. *prostrata*. Compounds **1**–**4**, particularly compound **2**, demonstrated significant suppression of IL-6 and TNF-α production, aligning with their robust binding affinities for critical inflammatory targets such as TNF, NFKB1, and EGFR, suggesting their anti-inflammatory actions are regulated through more than one target. These results are aligned with previous reports that found similar cytokine-suppressive effects in related natural compounds, which support the pharmacological importance of *E*. *prostrata* components. Our results provide a molecular basis for the traditional anti-inflammatory use of *E*. *prostrata* and highlight these compounds as potential candidates for the creation of novel therapies targeting inflammatory diseases.

## 4. Materials and Methods

### 4.1. Plant Materials and Separation of Compounds

*E*. *prostrata* whole plants were collected at Hoabinh provine (Vietnam) in August 2025. The fresh plants were removed from the impurity parts before drying at room temperature in shadow for five days. The dried materials were ground into powder before extracting with 75% EtOH by ultrasonic three times for 120 min, each bath. The crude extract (42 g) was suspended into water and partitioned with organic solvents according to increasing polarity, resulted in three fractions, n-hexane (3.8 g), EtOAc (29.6 g), and water residue (8.6 g). The EtOAc was further separated by using silica gel on an open column chromatography eluted by a gradient solvent system of methylene chloride/methanol/water (from 50/1/0.0001 to 1/2/0.1, *v*/*v*/*v*) to get 12 fractions (E1-E12). Subfraction E3 was loaded into an open column using ODS reverse silica eluted with a solvent system of acetone/water (1/1.2) to get **3** (4.0 mg) and **4** (5.4 mg). Subfraction E5 was separated by using a Sephadex column eluted with methanol/water (1/1) effort **1** (12.5 mg), **9** (3.0 mg), and **10** (1.2 mg). Subfraction E6 was purified by using a silica gel open column eluted with EtOAc/methanol/water (15/1) to get **2** (4.2 mg), **12** (1.9 mg), and **13** (1.1 mg). Subfraction E9 was separated by using a Sephadex column eluted with methanol/water (1/1) to get **8** (5.5 mg) and **11** (2.4 mg). Subfraction E11 was isolated by using an ODS reverse silica on an open column chromatography eluted with a solvent system of acetone/water (1/2) to get **5** (0.9 mg) and **7** (5.8 mg), and further purified using open column chromatography with silica gel eluting with methylene chloride/methanol (3/1) to obtain **6** (2.6 mg).

The spectroscopic data of isolated compounds were operated by using a JEOL JNM-AL 400 MHz spectrometer (JEOL, Tokyo, Japan), with chemical shifts reported as δ values (ppm) relative to TMS as the internal standard (measured in CD_3_OD). The HR-ESI-MS/MS confirmation was conducted using a Thermo Scientific Vanquish UHPLC system (Thermo Fisher Scientific, Sunnyvale, CA, USA) in conjunction with the Orbitrap Exploris 120 mass spectrometer system (SCIEX, Foster City, CA, USA).

### 4.2. Network Pharmacology

#### 4.2.1. Compound and Inflammation-Related Target Prediction

Chemical structures of isolated compounds (**1**–**13**) were inserted into the PubChem website (https://pubchem.ncbi.nlm.nih.gov/, accessed on 12 September 2025), and then their SMILEs were subjected to the Sea web service (https://sea.bkslab.org/, accessed on 13 September 2025) to get 331 compound targets by setting Human as the species criteria. Inflammation-associated targets were collected from the GeneCard database (https://www.genecards.org, accessed on 15 September 2025) by setting it as “Homo sapiens” and following a threshold > 5 to achieve 729 disease targets.

#### 4.2.2. Protein–Protein Interaction Network and Topological Analysis

Venn diagram was utilized to delineate the overlapping targets between compounds and inflammation-related targets. Subsequently, five overlapping targets were uploaded to the STRING webserver (https://string-db.org/, accessed on 16 September 2025). The selection of “Homo sapiens”, the default parameters (high confidence of 0.7 for confidence score and 5% for FDR stringency) were used to filter the findings. Output network was utilized as the String plugin in Cytoscape version 3.10.1 for visualization to establish the PPI network. Hub genes were determined by using the Cytohubba plugin in the Cytoscape program.

#### 4.2.3. Gene Ontology (GO) and the Kyoto Encyclopaedia of Genes and Genomes (KEGG) Pathways

The functional enrichment analysis of overlapping therapeutic targets was evaluated using the KEGG and GO enrichments. GO enrichment study reveals the biological mechanisms linked to principal therapeutic targets. KEGG enrichment analysis illustrates the functional pathway annotations of principal targets. Three GO (biological process, cellular component, and molecular function) were examined and illustrated using SRPLOT web tools (https://www.bioinformatics.com.cn/en; Shanghai Jiaotong University, Xiangya School of Medicine, Changsha, Hunan, China; accessed on 28 September 2025). Moreover, the KEGG pathway enrichment analysis was conducted using ShinyGO 0.82 (http://bioinformatics.sdstate.edu/go/; South Dakota State University, Brookings, SD, USA).

### 4.3. Prediction of Binding Affinities of Compounds and Target Proteins

AutoDock Tools 1.5.6 was utilized to assign partial charges to the protein and ligand, including the absent hydrogens, and establish the grid box parameters. The structures of the proteins [Protein (PDB ID): TNF (2AZ5) [27], AKT1 (1H10) [28], EGFR (5D41) [30], IL6 (1ALU) [30], NFKB1 (2O61) [29], and PTGS2 (5F1A) [45]] were acquired from the Protein Data Bank https://www.rcsb.org, accessed on 17 September 2025). The compound structures were obtained from PubChem (https://pubchem.ncbi.nlm.nih.gov; accessed on 18 August 2025). Prior to the addition of hydrogen atoms and Kollman charges, the proteins were devoid of water and heteroatoms. The structure of compound **6** was built by using the Chemdraw3D (Ver. 2016) before energy optimization using the Avogadro software (Ver.1.2.0) to save the output result as a pdb format. The Open Babel tool was utilized to convert the ligand into pdbqt format, and the torsion corrections along with Gasteiger charges were computed. Autodock 4.2.6 was performed with default docking parameters to predict the binding affinity of compounds to each protein. The native ligands of proteins such as 2AZ5, 5D41, and 5F1A were redocked, showing the RMSD values of 1.04, 1.60, and 0.09 Å, respectively. The results indicated the accuracy of the docking protocols. Other proteins, which did not contain native ligands [29,30] or dock to specific binding sites [28], were performed by following the methods reported in the previous studies. Finally, the docking analysis was visualized through Discovery Studio 2021 software.

### 4.4. Bioassays

#### 4.4.1. Cell Culture and Toxicity

Procell of Wuhan, China, was where we got the RAW264.7 cells. The cells were grown in DMEM media (Manufactured by Gibco, Grand Island, NY, USA), which was supplemented with 100 IU/mL penicillin G, 10% FBS, 100 µg/mL streptomycin, followed by incubating at 37 °C in a humidified environment with 5% CO_2_ and 95% air. Following a 1 h incubation of RAW264.7 cells (1 × 10^5^ cells/mL), each compound was introduced in the working solution. The viability of RAW 264.7 cells was assessed over a 24 h period utilizing an MTT reagent (Dojindo, Tokyo, Japan) at a wavelength of 540 nm.

#### 4.4.2. ELISA Assays

RAW264.7 cells (1 × 10^5^ cells/mL) were stimulated with 10 ng/mL LPS (Sigma-Aldrich, Saint Louis, MO, USA), with or without varying doses of substances (0, 10, 20, 50, or 100 µM), for 24 h at 37 °C in a 5% CO_2_ atmosphere. The culture supernatants were utilized for ELISA. Following the manufacturer’s instructions, BD OptEIATM ELISA kits (BD Biosciences, San Jose, CA, USA) were used to measure the levels of TNF-α and IL-6 that were produced in LPS-stimulated RAW264.7 cells. Each experiment was carried out three times.

### 4.5. Statistical Analysis

The results presented are expressed as the mean ± SD from three independent experiments. Statistical significance analysis was conducted utilizing analysis of variance (ANOVA), following Dunnet’s test from the GraphPad Prism software (version 8.0.1) (Boston, MA, USA).

## 5. Conclusions

In this study, we successfully separated and identified the first isolate, diosmin, along with twelve compounds, from *E*. *prostrata* by applying multiple chromatographic and spectroscopic techniques. These compounds exert their anti-inflammatory potential through signaling pathways, including IL-17 and TNF signaling pathways, by network pharmacology analysis. Molecular docking preliminarily verified their binding affinities of isolated compounds and core genes targeting inflammation. Cellular assays confirmed the anti-inflammatory effects of these compounds by suppressing TNF-α and IL-6 production in LPS-stimulated RAW264.7 cells, showing that flavonoids should be major active constituents that promote the anti-inflammatory effect of this plant. Our finding supported *E*. *prostrata* having anti-inflammatory properties by active constituents. Further studies are necessary to ascertain the interaction mechanism of the active components to clarify their anti-inflammatory efficacy.

## Figures and Tables

**Table 1:** Inhibitory effect of extract and fractions on inflammatory cytokine production.

Fraction	IL-6 (μg/mL)	TNF-α (μg/mL)
Ext.	55.68 ± 1.05	66.18 ± 3.16
H	71.00 ± 3.38	78.22 ± 4.87
E	43.24 ± 0.83	45.97 ± 2.01
W	82.02 ± 2.81	95.37 ± 1.96
Dexa.	1.85 ± 0.06	2.55 ± 0.08

Dexamethasone (Dexa.) was used as the positive control.

**Table 2:** Docking scores (kcal/mol) of isolated compounds (**1**–**13**) into core target proteins.

Compound	IL-6	TNF	AKT1	NFKB1	EGFR	PTGS2	Average Score
**1**	−4.51	−5.01	−4.85	−5.96	−6.23	−5.89	−5.41
**2**	−4.65	−5.63	−5.17	−6.50	−8.01	−7.80	−6.29
**3**	−4.91	−6.20	−5.03	−6.20	−7.91	−6.87	−6.19
**4**	−4.25	−5.65	−4.42	−5.89	−6.77	−7.03	−5.73
**5**	−3.45	−5.47	−3.41	−3.44	−2.13	−4.87	−4.27
**6**	−4.40	−5.20	−4.37	−5.54	−5.59	−4.49	−4.96
**7**	−4.37	−4.96	−4.09	−5.16	−4.91	−5.02	−4.75
**8**	−4.39	−4.59	−4.75	−5.44	−6.84	−5.44	−5.24
**9**	−4.32	−4.33	−4.43	−6.38	−6.29	−5.96	−5.29
**10**	−4.58	−4.35	−3.44	−5.82	−5.25	−5.49	−4.82
**11**	−3.48	−4.72	−3.51	−5.83	−5.13	−6.40	−4.85
**12**	−4.41	−4.37	−3.73	−4.99	−5.30	−5.56	−4.81
**13**	−4.34	−4.62	−3.55	−5.76	−5.02	−5.92	−4.87

Average score (kcal/mol) represents the arithmetic mean of docking scores for each compound across all six target proteins used to compare the overall binding tendencies among compounds.

**Table 3:** Anti-inflammatory effects of isolated compounds on IL-6 and TNF-α production.

Compound	IL-6 (μM)	TNF (μM)
**1**	5.66 ± 0.54	22.12 ± 1.18
**2**	13.24 ± 0.73	2.56 ± 0.08
**3**	9.89 ± 0.80	6.18 ± 0.03
**4**	25.45 ± 1.26	8.01 ± 0.11
**5**	88.71 ± 3.42	75.43 ± 2.60
**6**	95.26 ± 3.60	23.07 ± 0.84
**7**	80.56 ± 2.53	73.49 ± 4.04
**8**	17.45 ± 1.30	64.75 ± 3.07
**9**	37.86 ± 1.09	12.30 ± 1.06
**10**	79.91 ± 4.18	98.52 ± 2.17
**11**	32.55 ± 0.98	38.76 ± 1.29
**12**	36.28 ± 2.51	44.37 ± 1.04
**13**	23.81 ± 0.45	52.67 ± 2.19
**Dexa.**	3.62 ± 0.05	2.20 ± 0.09

## Data Availability

Data are contained within the article and Supplementary Materials.

## Supplementary Materials

The following supporting information can be downloaded at: https://www.mdpi.com/article/10.3390/ph18111653/s1, Table S1: Spectroscopic data of compound **5**; Figure S1. Cytotoxic effect of extract (Ext.) and fractions on viability of RAW264.7 cells; Figure S2. IL-17 signaling pathway; Figure S3. TNF signaling pathway; Figure S4: Visualization of interactions of compounds and IL-6 protein; Figure S5: Visualization of interactions of compounds and AKT1 protein; Figure S6: Visualization of interactions of compounds and EGFR protein; Figure S7: Visualization of interactions of compounds and NFκB1 protein; Figure S8: Visualization of interactions of compounds and PTGS2 protein; Figure S9. Cytotoxic effects of isolated compounds (**1**–**13**) on viability of RAW264.7 cells.
