# Supplementary material for: Identification and Integrative Discovery of Anti-Inflammatory Compounds Isolated from Eclipta prostrata (L.) L. by Network Pharmacology, Molecular Docking, and In Vitro Evaluation
Source: Pharmaceuticals (Basel). 2025 Nov 1;18(11):1653. doi: 10.3390/ph18111653 (PMC12655162; doi:10.3390/ph18111653)
Supplement: Supplementary file 1 [file pharmaceuticals-18-01653-s001.zip › pharmaceuticals-3919323-supplementary.pdf]

Identification and Integrative Discovery of Anti-Inflammatory Compounds Isolated from *Eclipta prostrata* (L.) L. by Network Pharmacology, Molecular Docking, and In Vitro Evaluation

Cao Van Anh [1], Nguyen Ngoc Linh [2] and Phuochien Phan [3,4,*]

[1] Institute of Pharmaceutical Education, Vietnam Military Medical University, Hanoi 100000, Vietnam; caovananh12a1@gmail.com
[2] Institute of Medicine and Pharmacy, Thanh Do University, Hoai Duc, Hanoi, Vietnam; nnlinh@thanhdouni.edu.vn
[3] Institute of Applied Science and Technology, Van Lang School of Technology, Van Lang University, Ho Chi Minh City, Vietnam
[4] Faculty of Applied Technology, Van Lang School of Technology, Van Lang University, Ho Chi Minh City, Vietnam
* Correspondence: hien.pp@vlu.edu.vn; Tel.: +84-2871099237

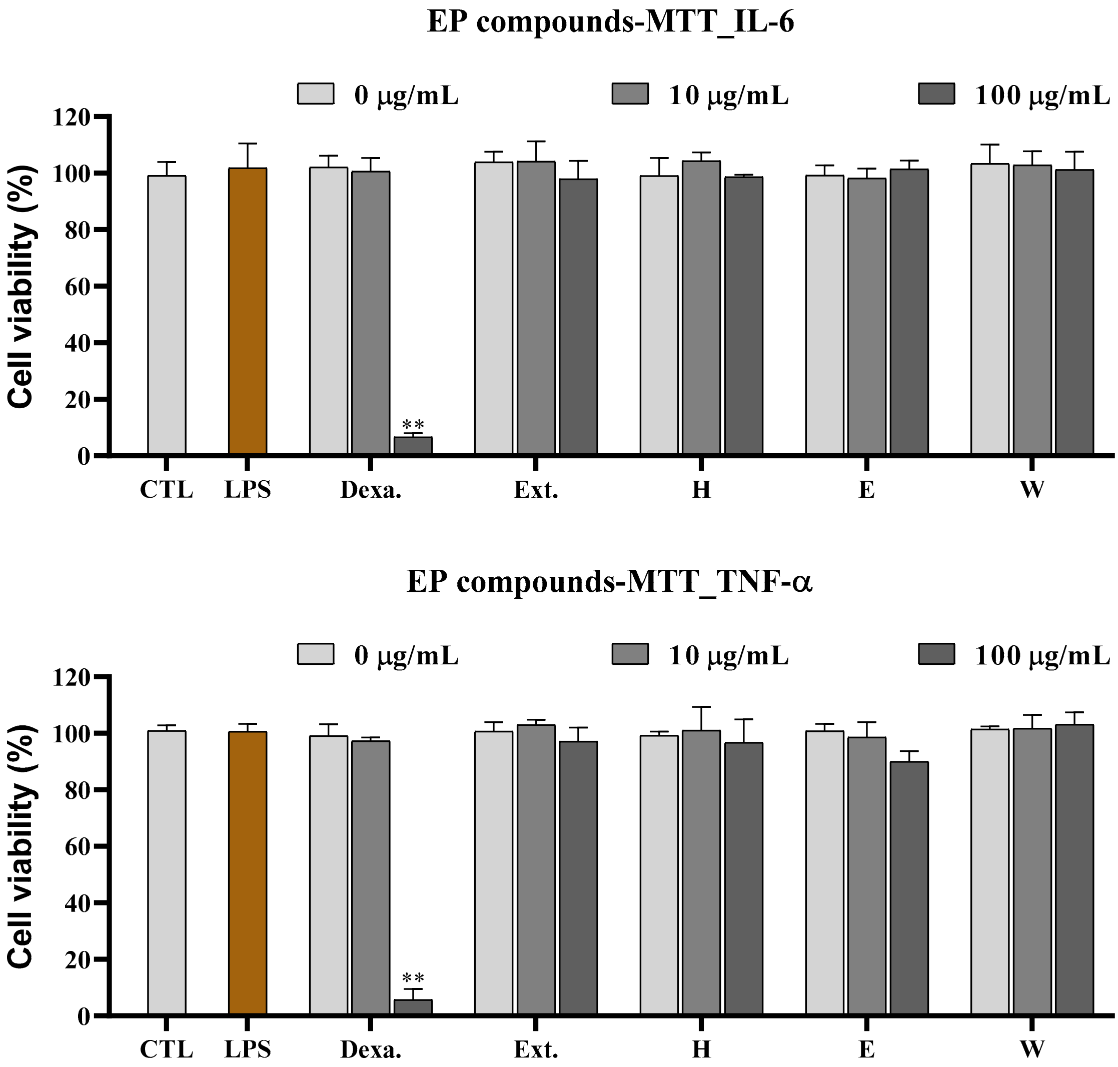

Figure S1. Cytotoxic effect of extract (Ext.) and fractions on viability of RAW264.7 cells.

Table S1. ^{1}H (400 Mhz) and ^{13}C NMR (100 Mhz) spectroscopic data of compound **5**.

Position	**5 (DMSO-d_6)**	
	**δ_H mult (400 MHz, *J*=Hz)**	**δ_C (100 MHz)**
2		164.2
3	6.82 (1H, s)	103.8
4		182.0
5		161.2
6	6.46 (1H, d, *J* = 2.2 Hz)	99.6
7		162.9
8	6.76 (1H, d, *J* = 2.2 Hz)	94.8
9		156.9
10		105.5
1'		122.9
2'	7.45 (1H, d, *J* = 2.3 Hz)	113.2
3'		146.8
4'		151.3
5'	7.13 (1H, d, *J* = 8.8 Hz)	112.2
6'	7.57 (1H, dd, *J* = 8.8, 2.3 Hz)	119.0
4'-OCH_3	3.87 (3H, s)	55.8
Glu		
1''	5.08 (1H, d, *J* = 7.4 Hz)	99.9
2''	3.28 (d, 8.8)	73.1
3''	3.27 (m)	76.3
4''	3.65 (m)	70.3
5''	3.74 (m)	76.0
6''	3.84 (d, 11.8, 1.7)	66.1
	3.44 (m)	
Rha		
1'''	4.54 (1H, d, *J* = 1.7 Hz)	100.5
2'''	3.65	70.3
3'''	3.40	68.1
4'''	3.15	72.0
5'''	3.46	70.7
6'''	1.07 (3H, d, *J* = 6.0 Hz)	17.8

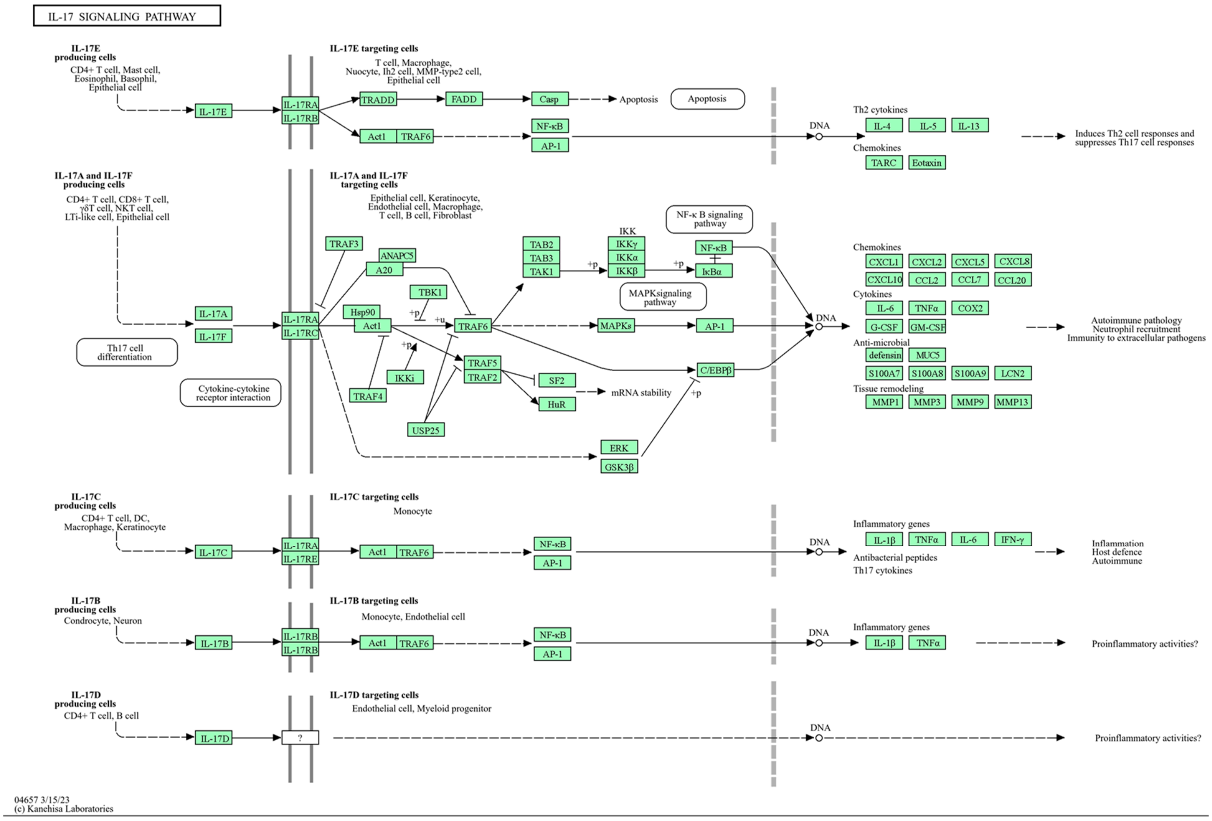

Figure S2. IL-17 signaling pathway.

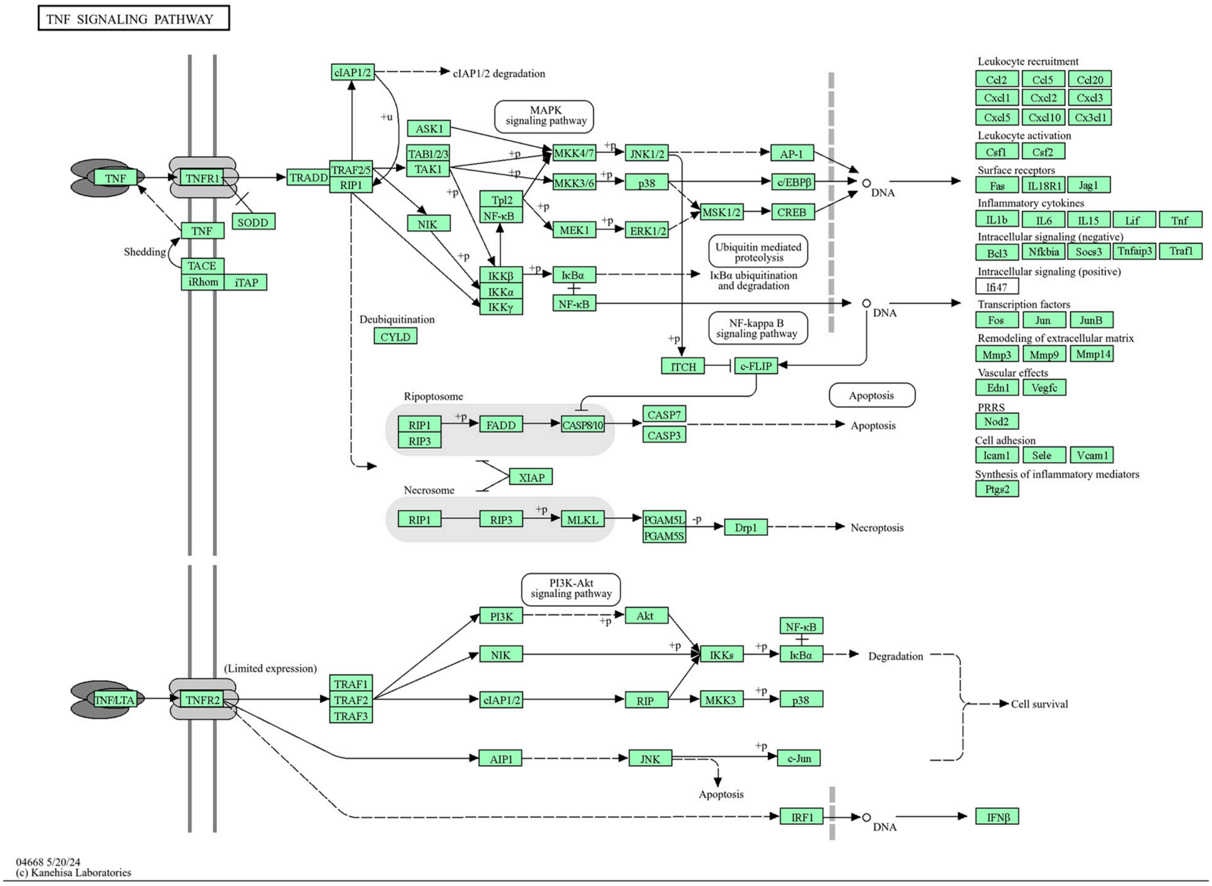

Figure S3. TNF signaling pathway.

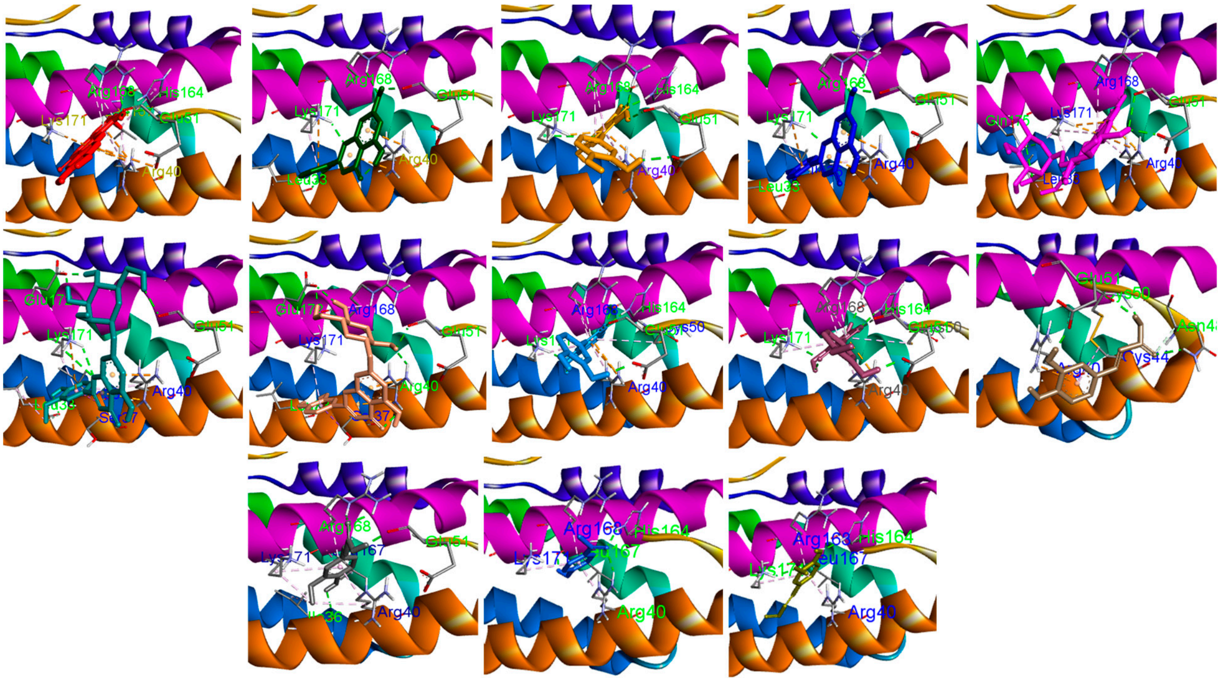

Figure S4. Visualization of **1** (red), **2** (green), **3** (orange), **4** (blue), **5** (magenta), **6** (cyan), **7** (wheat), **8** (light blue), **9** (purple), **10** (sand), **11** (grey), **12** (marine), **13** (limon) interacting with IL6 protein. The compound interacts with residues (green) via hydrogen bond formation.

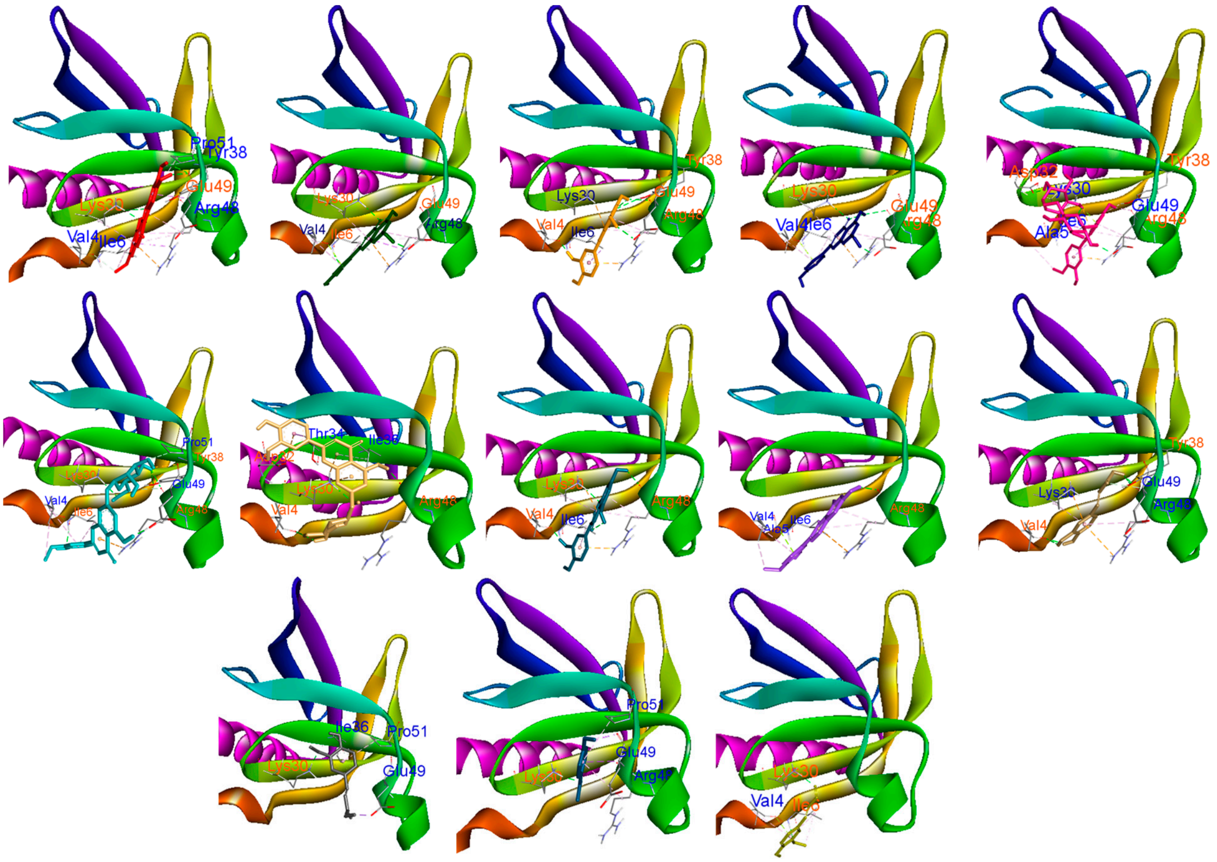

Figure S5. Visualization of **1** (red), **2** (green), **3** (orange), **4** (blue), **5** (magenta), **6** (cyan), **7** (wheat), **8** (light blue), **9** (purple), **10** (sand), **11** (grey), **12** (marine), **13** (limon) interacting with AKT1 protein. Hydrogen bonds formed between the compound and key residues (orange) mediate their interaction.

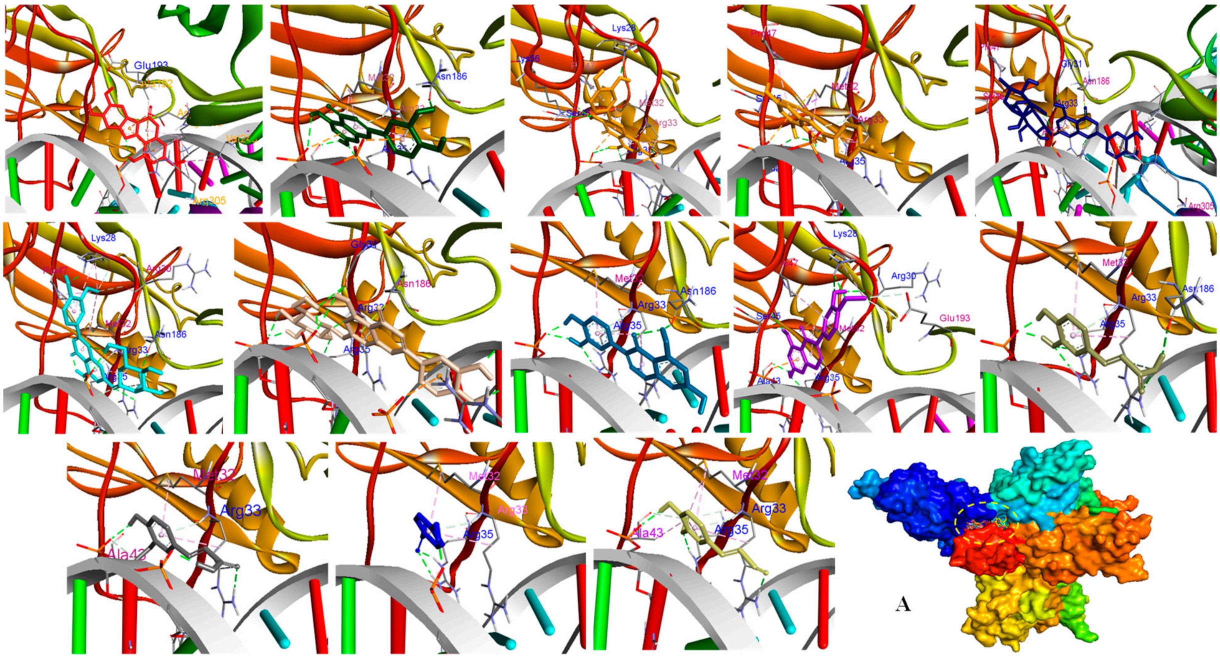

Figure S6. Visualization of **1** (red), **2** (green), **3** (orange), **4** (blue), **5** (magenta), **6** (cyan), **7** (wheat), **8** (light blue), **9** (purple), **10** (sand), **11** (grey), **12** (marine), **13** (limon) interacting with NFκB1 protein. Hydrogen bonds formed between the compound and key residues (blue) mediate their interaction. (A) Compounds **1–13** docked in the protein binding site.

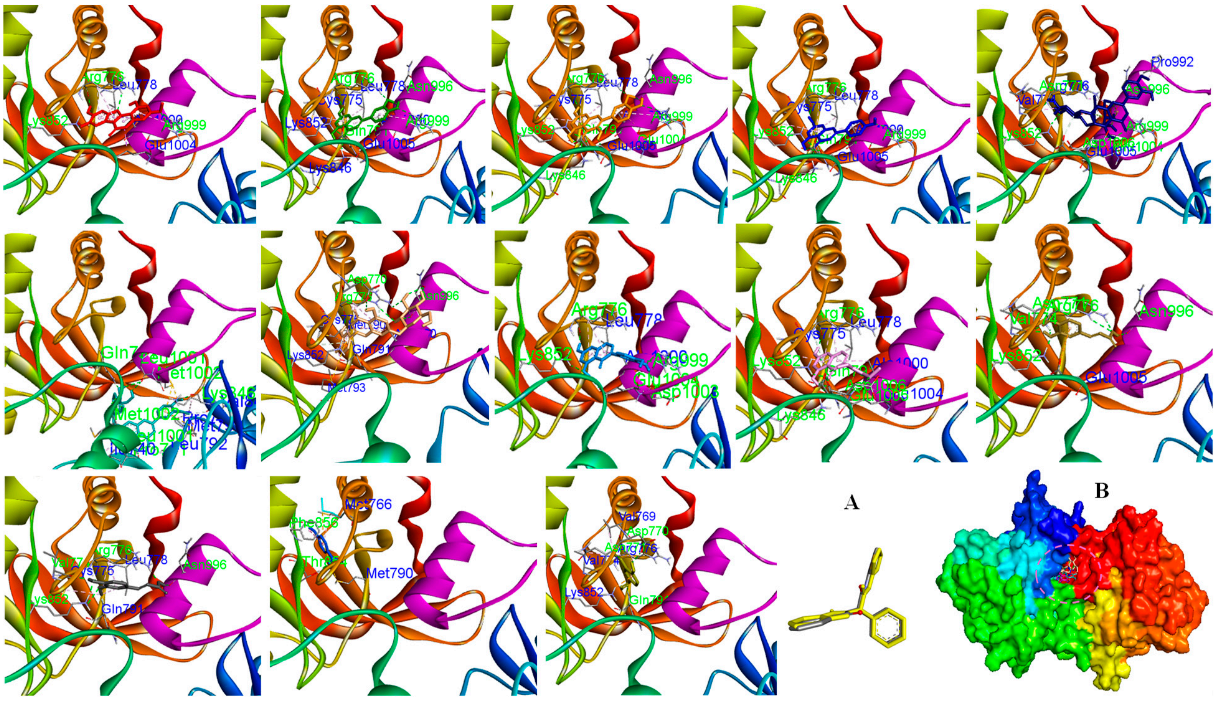

Figure S7. Visualization of **1** (red), **2** (green), **3** (orange), **4** (blue), **5** (magenta), **6** (cyan), **7** (wheat), **8** (light blue), **9** (purple), **10** (sand), **11** (grey), **12** (marine), **13** (limon) interacting with EGFR protein. Hydrogen bonds formed between the compound and key residues (green) mediate their interaction. (A) Superposition of original and redocked native ligand. (B) Compounds **1–13** docked in the protein binding site.

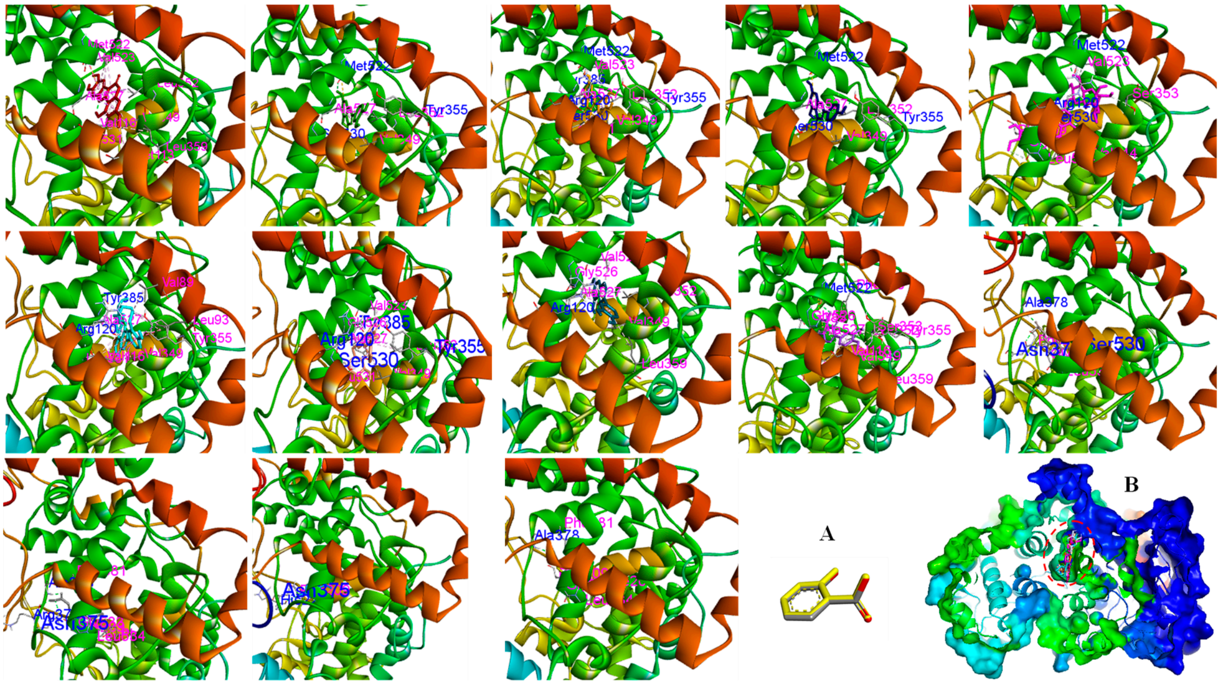

Figure S8. Visualization of **1** (red), **2** (green), **3** (orange), **4** (blue), **5** (magenta), **6** (cyan), **7** (wheat), **8** (light blue), **9** (purple), **10** (sand), **11** (grey), **12** (marine), **13** (limon) interacting with PTGS2 protein. Hydrogen bonds formed between the compound and key residues (blue) mediate their interaction. (A) Superposition of original and redocked native ligand. (B) Compounds **1–13** docked in the protein binding site.

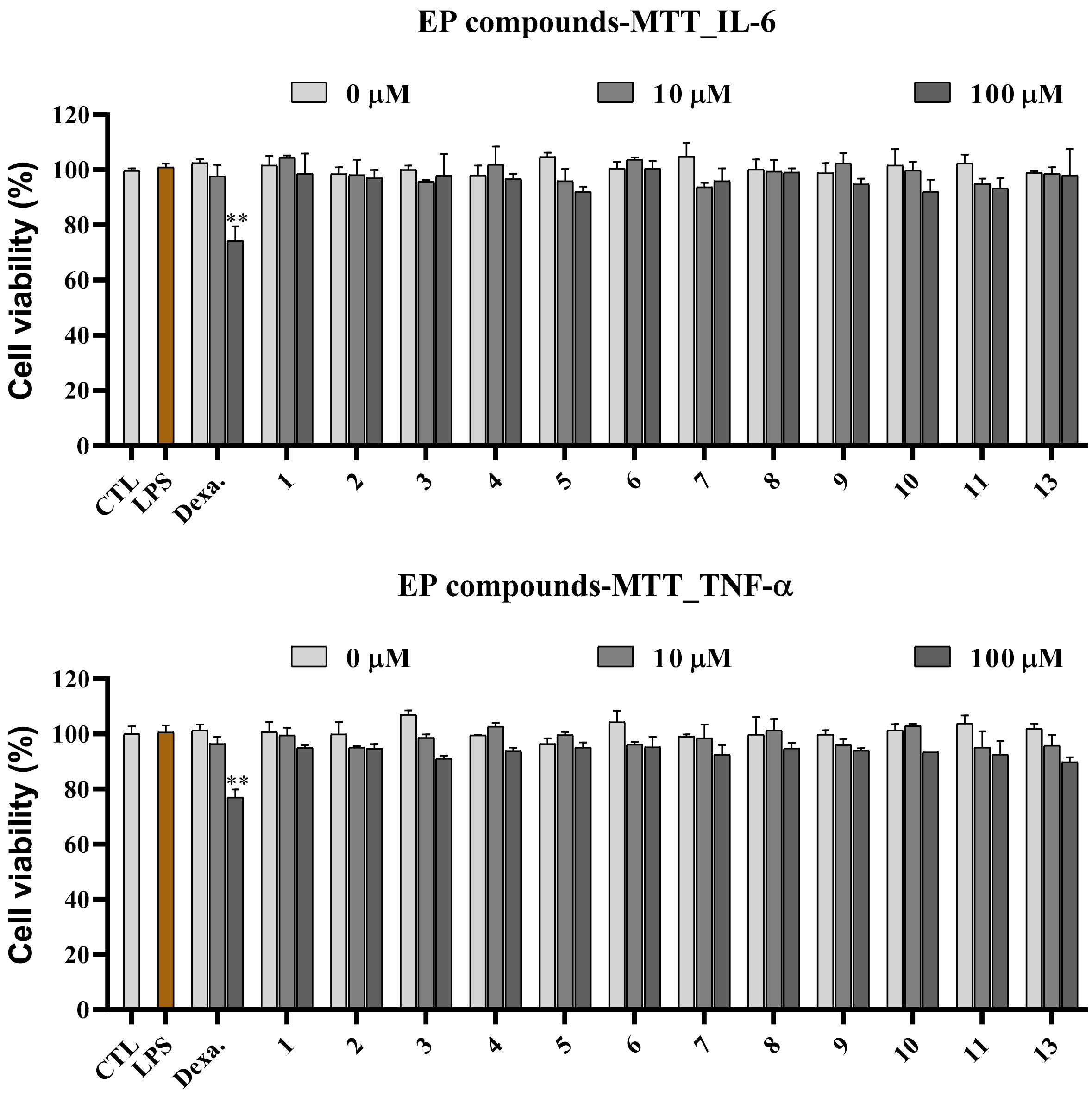

Figure S9. Cytotoxic effects of isolated compounds (**1**–**13**) on viability of RAW264.7 cells.
